# Axonal Kainate Receptors Modulate the Strength of Efferent Connectivity by Regulating Presynaptic Differentiation

**DOI:** 10.3389/fncel.2016.00003

**Published:** 2016-01-20

**Authors:** Prasanna Sakha, Aino Vesikansa, Ester Orav, Joonas Heikkinen, Tiina-Kaisa Kukko-Lukjanov, Alexandra Shintyapina, Sami Franssila, Ville Jokinen, Henri J. Huttunen, Sari E. Lauri

**Affiliations:** ^1^Neuroscience Center, University of HelsinkiHelsinki, Finland; ^2^Department of Biosciences, University of HelsinkiHelsinki, Finland; ^3^Departments of Materials Science and Engineering, Aalto UniversityEspoo, Finland; ^4^Department of Veterinary Biosciences, University of HelsinkiHelsinki, Finland

**Keywords:** glutamate receptor, kainate receptor, presynaptic, glutamate release probability, microfluidic, synaptogenesis

## Abstract

Kainate type of glutamate receptors (KARs) are highly expressed during early brain development and may influence refinement of the circuitry, via modulating synaptic transmission and plasticity. KARs are also localized to axons, however, their exact roles in regulating presynaptic processes remain controversial. Here, we have used a microfluidic chamber system allowing specific manipulation of KARs in presynaptic neurons to study their functions in synaptic development and function *in vitro*. Silencing expression of endogenous KARs resulted in lower density of synaptophysin immunopositive puncta in microfluidically isolated axons. Various recombinant KAR subunits and pharmacological compounds were used to dissect the mechanisms behind this effect. The calcium permeable (Q) variants of the low-affinity (GluK1–3) subunits robustly increased synaptophysin puncta in axons in a manner that was dependent on receptor activity and PKA and PKC dependent signaling. Further, an associated increase in the mean active zone length was observed in electron micrographs. Selective presynaptic expression of these subunits resulted in higher success rate of evoked EPSCs consistent with higher probability of glutamate release. In contrast, the calcium-impermeable (R) variant of GluK1 or the high-affinity subunits (GluK4,5) had no effect on synaptic density or transmission efficacy. These data suggest that calcium permeable axonal KARs promote efferent connectivity by increasing the density of functional presynaptic release sites.

## Introduction

Ionotropic glutamate receptors are well known for their critical roles in mediating and modulating excitatory neurotransmission in the brain. Kainate type of glutamate receptors (KARs) are composed of five different subunits that can be grouped into low-affinity (GluK1–3) and high-affinity (GluK4–5) subtypes. The subunit composition of KAR controls their subcellular targeting and channel properties, thus defining the physiological role of the receptor in the neuronal network. Postsynaptic KARs typically mediate slow synaptic currents well suited for integration of synaptic responses, while both facilitatory and inhibitory actions of presynaptic KAR on transmitter release have been described (reviewed by Jane et al., 2009; Contractor et al., 2011; Lerma and Marques, 2013). Apart from the ionotropic effects, KARs modulate neuronal functions, via G-protein coupled signaling, especially during the ‘critical period’ of circuit development in the hippocampus (Lauri and Taira, 2011).

Increasing evidence suggests that KARs and in particular, presynaptically localized KARs have specific developmentally restricted functions that are not directly related to the fast synaptic signaling. KARs influence neurite outgrowth and morphological maturation of neurons (Ibarretxe et al., 2007; Joseph et al., 2011; Marques et al., 2013), regulate mobility of the axonal filopodia (Chang and De Camilli, 2001; Tashiro et al., 2003) and mobilization of the synaptic vesicles in the growth cones (Gelsomino et al., 2013). At the stage when a synaptic connection is formed and is already functional, presynaptic KARs are tonically active and inhibit vesicle release; this G-protein dependent signaling regulates the short-term dynamics of transmission (Lauri et al., 2006; Clarke et al., 2014) and is suggested to have an important role in activity-dependent fine-tuning of the connectivity in the hippocampus (Lauri and Taira, 2011).

Although it is established that impaired KAR activity is associated with delayed development of glutamatergic synaptic transmission (Marchal and Mulle, 2004; Vesikansa et al., 2007; Lanore et al., 2012), direct evidence on the role of KARs in synaptogenesis is lacking. Here, we have used a microfluidic platform allowing specific genetic control of protein expression in presynaptic neurons in combination with optogenetic stimulation and electrophysiological recordings (Jokinen et al., 2013), to study the role of presynaptic KARs in synaptic development and function at high resolution *in vitro*. Our data reveals that the axonal calcium permeable KARs coordinate presynaptic differentiation to enhance the strength of upcoming synaptic connection.

## Materials and Methods

### RT-qPCR

Total RNA was isolated from two independent cultures at DIV7 and DIV14 using RNeasy Micro Kit (Qiagen) with an on-column DNase digestion step. The concentration and the purity (A_260/280_) of RNA were measured using the NanoDrop (Thermo Scientific). cDNA was synthesized from 1 μg of total RNA using RevertAid First Strand cDNA synthesis Kit (Thermo Scientific) with oligo(dT)_18_ primer. External standards for absolute quantification were made by PCRs carried out with real-time PCR primers (**Table 1**), Phusion High-Fidelity DNA polymerase (Thermo Scientific), and cDNA template. PCR-amplified regions were verified by agarose gel electrophoresis and sequencing. The PCR products were purified. The DNA concentration was measured and copy numbers (N) were calculated for each external standard. aqPCR was performed in a CFX96 Real-Time PCR Detection System (Bio-Rad) using Maxima SYBR Green qPCR Master Mix (Thermo Scientific), 200 nM real-time PCR primers, 5 μl template (1:50 diluted cDNA or diluted standard [serially diluted from 10^6^ to 10^2^ (copies/5 μl)]. Each run was completed with a melting curve yielding only one sharp peak of specific product. All samples were analyzed in duplicate. The initial copy numbers of a sample was obtained by relating the Ct of the sample to a standard curve plot. Gene expression differences in DIV7 and DIV14 were also confirmed by relative qPCR, using *Gapdh* and *Rpl19* as reference genes and 2^-ΔΔCt^ method relative to DIV7.

**Table 1:** Real-time PCR primers.

Target	Forward	Reverse	Size (bp)
GluK1	ATGTGACGCAGAGGAACTGC	GCAGTTGAAGAATGGCAATCG	126
GluK2	GTTTGTTACACAGCGGAACTG	CAGCTGAAGAATTGCTATGGTG	127
GluK3	CATCGATTCCAAGGGCTACG	CGCCACCACTTCTCCTTCAT	126
GluK4	GACACCAAGGGCTATGGGAT	ACCACTTCCGCTTCAGAATC	118
GluK5	AGTACGGCACTATCCACGCT	CTCCTCTGTGCTCTTGACGA	128
*Gapdh*	CAGTGCCAGCCTCGTCTCATA	TGGTAACCAGGCGTCCGATA	79
*Rpl19*	ATGAGTATGCTTAGGCTACAGA	GCATTGGCGATTTCGTTGGT	104

### Lentiviral Constructs

Plasmids encoding epitope-tagged kainate receptor subunits were constructed as described (Vesikansa et al., 2012). The epitope tagged constructs were subcloned into lentiviral transfer vector under synapsin-1 or CMV promoter (pLen-Syn1/CMV) (**Table 2**). All constructs were verified by restriction mapping and by sequencing of PCR-amplified regions. The appropriate size of the encoded recombinant proteins was confirmed by Western blot of transfected HEK293T cells.

**Table 2:** List of constructs used.

GFP/pLen-d-Syn1
GFP/pLen-CMV
GluK1 2c(Q)-flag/pLen-CMV
GluK1 2c(Q)-myc/pLen-d-Syn1-EGFP
GluK1 2c(R)-myc/pLen-d-Syn1-EGFP
GluR2(Q)-myc/pLen-CMV
GluR3(Q)-myc/pLen-CMV
GluK4-myc/pLen-CMV
GluK5-myc/pLen-CMV
GluA2-myc/pLen-CMV
Scrambled shRNA pLKO.1/Syn1-EGFP
GluK2 shRNA(2-2) pLKO.1/Syn1-EGFP
GluK5 shRNA(5-1) pLKO.1/Syn1-EGFP
pLen-Syn1(mock)
pLen-CMV(mock)
ChR2(H134R)/pLen-Syn1-EYFP

Five different shRNA sequences against rat GluK2 and GluK5 in pLKO.1 vector (obtained from Sigma–Aldrich) were tested for their efficiency to suppress expression of GluK2-myc and GluK5-myc in HEK293T cells. Cells were co-transfected with 400 ng of Myc-GluK1-5/pLenCMV + 1600 ng of the shRNA (Grik2-1,2,3,4,5) using FuGENE HD. After 48 h transfections, cells were lysed in Laemmli buffer (5–10 min at +95°C). Western blots were stained with anti-myc antibodies (Rabbit Polyclonal, Upstate 06-549; 1:1000), followed by anti-actin staining of filters to verify equal protein loading. The construct with the best efficiency and specificity to knock down expression of the target protein in the heterologous system was selected and subcloned into a modified pLKO.1 vector where the puromycin resistance cassette was replaced with GFP under the synapsin-1 promoter (pLKO.1-syn1-EGFP). The selected target sequence for rat GluK2 (GRIK2, 888–912) was CTGCCAGCTGATACCAAAGAT and for rat GluK5 (GRIK5, 752–776) CCGGATCCTCAAGTCCTTTAA.

Lentiviral particles were produced in HEK293T cells as described (Vesikansa et al., 2012). HEK293T cells were seeded at the density of 3 × 10^6^ on 10 cm^2^ plates and transfected with Fugene6 (Roche Applied Science) on the following day using 0.75 μg envelope-coding plasmid pMD.G, 2.25 μg packaging plasmid psPAX2, and 3 μg of transfer vector (derivate of the original pHR’ backbone). Medium containing the viral particles was harvested 48 h post-transfection, cleared of debris by low-speed-centrifugation and concentrated immediately by ultracentrifugation (50,000 × *g*, 2 h at +4°C) or with PEG-it^TM^ virus precipitation solution (System Biosciences). Pellets were suspended in DMEM or PBS in 1/100–1/200 of the original volume. The titers of the lentiviral stocks were determined with ELISA assay (Aalto Bio Reagents) and were typically 1 × 10^7^–1 × 10^8^ transducing units/ml.

After production of lentiviral particles, the shRNA vectors were then further tested in hippocampal neurons where KAR subunits are endogenously expressed. The neurons were infected with the shRNA encoding lentiviral vectors at DIV5, and lysed in Laemmli buffer at DIV12. Western blots were stained with anti-GluK2/3 (rabbit anti-GluR6/7 clone NL9, Millipore, 1:1000) or anti-GluK5 (rabbit anti-KA2, Millipore, 1:1000), followed by anti-actin staining of filters to verify equal protein loading.

### Microfluidic Culture and Transduction

Hippocampi were dissected from 17/18-day-old rat embryos, treated with papain (500 μg/ml) and mechanically triturated to produce a single-cell suspension. Custom made microfluidic culture plates with 34 parallel axonal tunnels 2 mm in length and 7.5 μm in width (Jokinen et al., 2013) were fixed onto poly-L-Lysine coated coverslips. The reservoirs were filled with 180 μl of neurobasal media, containing 2% B27 supplement, 0.5 mM L-glutamine and 1% (v/v) penicillin-streptomycin (all from Life Technologies), and placed in the incubator (37°C, 5% CO_2_). Before plating the cells, 150 μl of media was removed from the chambers and RHN were seeded with the density of 13–25000 cells per reservoir. Cells were then allowed to settle for 10 min in the incubator and then full media was added making the final volume 180 μl. Then, 3/4 of the old media was replaced every third day with fresh warm (+37°C) fully supplemented media.

Neurons on one cell reservoir were infected with lentiviruses at 3 DIV or at DIV 7 (shRNA constructs) by adding 0.5–2 μl of concentrated lentiviruses for 20–25000 cells. During lentiviral infection, smaller media volume was maintained in the infected reservoir to assure asymmetric transduction (Jokinen et al., 2013). For pharmacological experiments, the culture media was supplemented with ACET (200 nM), KT5720 (1 μM) or bisindolylmaleimide VII acetate (BIS, 0.5 μM) from DIV3–4 onward. The control samples were treated with corresponding v/v of DMSO. Transduced neurons were analyzed at 15–18 DIV. Cultures where less than 20% of the tunnels contained axons crossing to the other reservoir were excluded from analysis.

### Immunostainings, Imaging, and Image Analysis

For immunostainings, cells were washed with phosphate-buffered saline (PBS, pH 7.4) and fixed with 4% PFA in PBS for 35–45 min at room-temperature (RT). Coverslips containing fixed cells were gently detached from the microfluidic chamber, and washed with PBS several times. Fixed cells were treated with blocking buffer containing 5% goat serum, 1% BSA, 0.1% gelatin, 0.1% Triton X-100, 0.05% Tween-20 in 1X PBS. Primary antibodies (mouse anti-phosphoTau [Ser396(PHF13)] 1:1000, Cell Signaling Technology; guinea pig anti-synaptophysin-1 1:1000, Synaptic Systems; rabbit anti-Myc, 1:1500, Millipore and mouse anti-flag 1:1500, Sigma–Aldrich; anti-mouse-PSD-95, 1:500, BD Transduction Laboratories) were added in blocking solution and cells were incubated overnight with shaking at +4°C. Cells were then washed three times with PBS before incubation with secondary antibodies (AlexaFluor 405 goat anti-mouse, AlexaFluor 488 goat anti-rabbit, AlexaFluor 405 goat anti-mouse, AlexaFluor 568 goat anti-guineapig; Molecular Probes, all 1:2000 dilutions) for 1.5-2 h RT. After washing twice, the coverslips were mounted using ProLong Gold Antifade (Life Technologies) reagent.

Confocal images were taken with MP Leica TCS SP5 confocal microscope (HPX PL APO 63× 1.30 objective), LSM Zeiss700 confocal microscope (I LCI Plan-Neofluar 63×/1.3 Imm Korr DIC M27 objective) or LSM Zeiss 710 confocal microscope (alpha Plan-Apochromat 63×/1.46 Oil Korr M27 objective). Control samples (GFP or mock shRNA) were used to optimize imaging parameters which were kept constant for all the samples within the culture batch. Image containing 12–16 fluorescent serial stacks (optimal stack interval of 0.36–0.4 μm) was compiled by maximum intensity projection. Axons were imaged between <0.2 mm (proximal part), 0.6–1 mm (mid part) or >1.8 mm (distal part) of the tunnel openings from the infected side of the microfluidic chamber. Altogether, two to three independent chambers from at least two to three independent cultures per condition were analyzed.

Leica LAS AF lite, ZEN Blue, ZEN Black, and Adobe Photoshop software were used for image analysis. Infected axons were identified with the myc/flag staining or GFP expression (green channel), while blue channel (405 nm) stained with pTau was used for visualizing all axons present in tunnels. In some experiments, far red (magenta) channel was used for imaging PSD-95. One infected axon per tunnel was manually traced for analysis using free shape curve drawing tool. Only those processes that could be reliably traced as individual axons were included in the analysis. Synaptophysin1 positive puncta (568 nm, red channel) were counted from between 170 and 245 μm length of axon in different parts of tunnels. Number of puncta within clusters was estimated with the reference size of isolated puncta. Pearson’s coefficient in colocalization voxels of Myc-GluK and Synaptophysin-1 was calculated using Imaris software. The data is expressed as the average density/μm axon length, normalized to the corresponding value in control (GFP or mock shRNA) expressing cultures from the same batch.

### Electrophysiology and Optogenesis

For electrophysiological recordings, the neurons were placed in a submerged recording chamber (Luigs and Neumann, slice mini chamber I) mounted on Olympus BX51 fluorescence microscope. The chambers were constantly perfused with Artificial Cerebrospinal Fluid containing (mM) 110 NaCl, 5 KCl, 10 HEPES, 10 glucose, 2 CaCl_2_, and 1 MgSO_4_ (250 mOsm, pH 7.4) at +29–30°C. Whole cell recordings were performed with glass electrodes (resistance 4–7 MΩ) filled with solution containing (mM) 115 CsMeSO_4_, 10 HEPES, 5 EGTA and 5 MgATP and 5 QX-314 (pH 7.2, Osm 240) using multiClamp700B amplifier (Molecular Devices). After obtaining whole cell access blue light-evoked responses were recorded under voltage clamp at –70 mV. OptoLED light source (Cairn Research Ltd., UK) with 470 nm LED connected to the Olympus microscope was used for light stimulation (5 ms pulse). pClamp software was used for data collection and analysis.

The success rate, mean amplitude and potency (amplitude of successful responses) was calculated from 50 consecutive trials in each recorded cell. A success was defined as a negative deflection in the recorded signal with amplitude at least two times the average noise level, the peak within 3 ms from the onset of light pulse. Recordings with <2 successes were not included in the analysis. The data is presented as mean ± SEM for the number of cells indicated, and normalized to the level of corresponding control (GFP expressing) cells.

### Statistical Analysis

All the statistics are calculated from the raw data. Normal distribution of the data was tested using Shapiro–Wilk test, followed by ANOVA, Student’s two-tailed *t*-test or Mann–Whitney test of statistical significance.

### Electron Microscopy

For electron microscopy (EM), cultures were fixed in 2% glutaraldehyde +2% PFA in 0.1 M sodium cacodylate (NaCac) buffer, pH 7.4 for 20–30 min and washed twice with NaCac buffer before coding the samples for further processing. Samples were osmicated in 1% OsO_4_ (in 0.1 M NaNac) + K4(Fe(CN)6; 15 mg/ml) for 1h followed by 2X 3 min wash in 0.1 M NaCac buffer and subsequent 3X washes in distilled water (DW). Samples were blocked in 1% UA (0.3 M sucrose in DW) for 1 h in +4°C followed by 3X washes in DW (3 min each). Samples were then dehydrated once in 70% and 96% EtOH and twice with absolute alcohol for 1 min. Coverslips were dipped in acetone and placed on an aluminum plate and immediately covered by Epon. Beem capsule filled with Epon was placed upside down on top of the cells and incubated for 2 h RT and then baked at 60°C for 14 hr. Samples were transferred directly from oven to hot plate and coverslip was carefully removed. Epon capsule with embedded neurons were cut with diamond knife in 100 nm section thickness and selected sections were transferred to grids. Grids were placed on silicon grid holder and washed 3X with Milli Q (MQ) water before incubating with 0.5% uranyl acetate for 30 min and washed 3X with MQ water followed by incubation in 80 mM lead citrate for 1.2 min. Post stained grids were washed 3X with MQ water and dried on a filter paper.

Joel 1400 transmission electron microscope was used for imaging with 4000×–6000× magnification. Synaptic active zones were imaged from at least three serial grids for each blind-coded sample. ImageJ software was used to measure the length of active zones of synapses with visible or intact synaptic cleft and an average of 114 ± 38 synapses were analyzed per sample. After image analysis, samples were decoded to pool the data. Represented data is from two samples of two independent cultures.

## Results

### Asymmetric Manipulation of KAR Expression in Microfluidic Cultures

**FIGURE 1:**
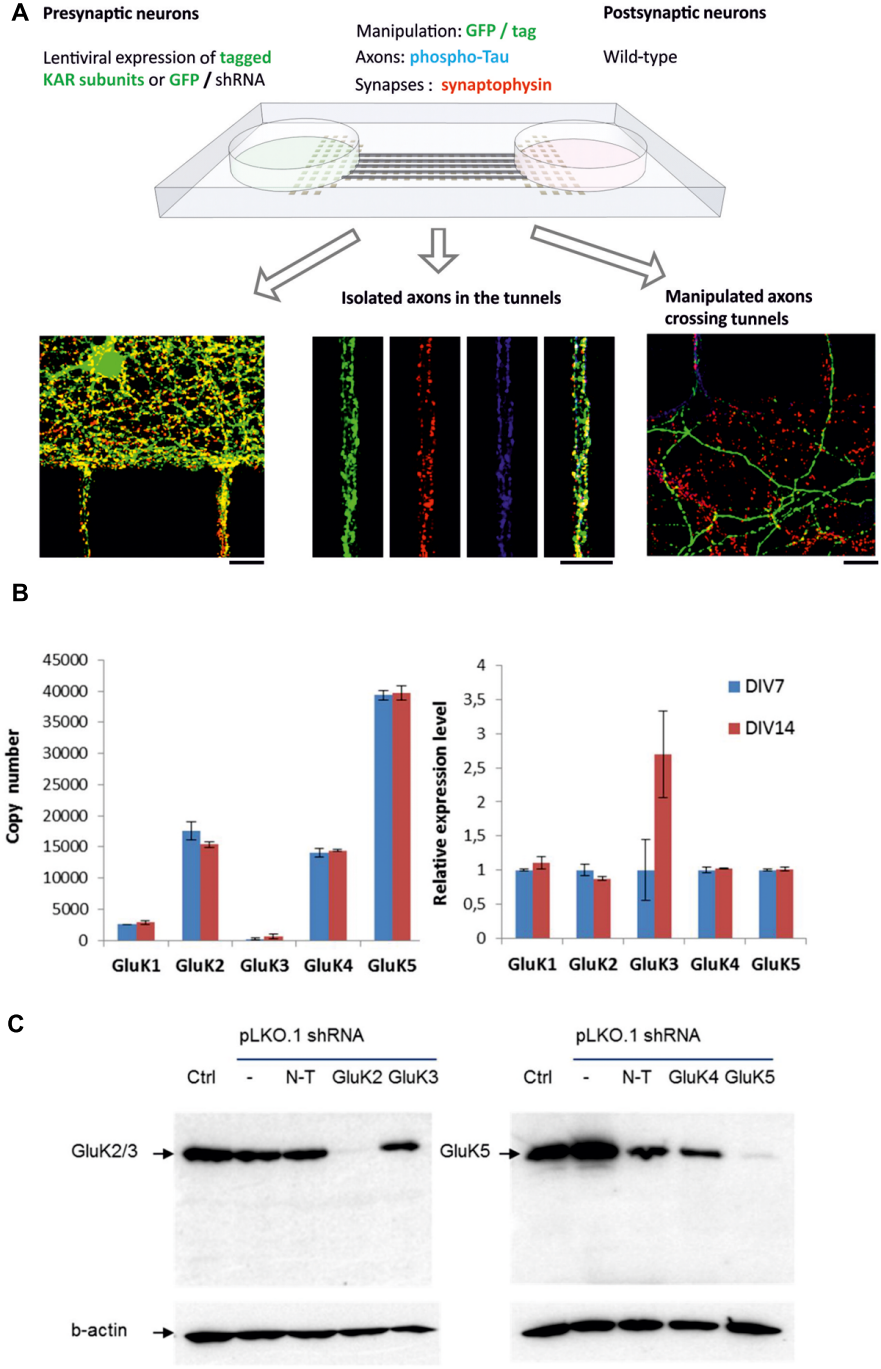
**The experimental setup and validation of the system. (A)** Schematic model of the microfluidic experimental system. Primary hippocampal neurons are grown in two reservoirs, connected by narrow tunnels allowing axon growth. On one side of the chamber, kainate receptor expression is manipulated using lentiviral vectors. The mid part of the tunnel contains isolated axons coming from both sides of the chamber, while on the uninfected side manipulated axons crossing the tunnels make contact to wild-type dendrites. Manipulated axons were identified with GFP expression and immunostaining against myc/flag tag (green). Immunostaining against synaptophysin (red) and phospho-Tau (blue) was used to visualize synaptic vesicle clusters and axons, respectively. Scale bar 15 μm. **(B)** RT-qPCR data illustrating the expression of various KAR subunits in the microfluidic cultures at DIV7 and DIV14. The graph on the left depicts the copy number of each mRNA, calculated using external standards. Gene expression differences in DIV7 and DIV14 were also analyzed by relative qPCR, using *Gapdh* and *Rpl19* as reference genes and 2^-ΔΔCt^ method relative to DIV7 (right). **(C)** Validation of the lentiviral shRNA constructs against GluK2 and GluK5 in primary hippocampal neurons. A western blot illustrating that the GluK2 shRNA, but not the non-target or GluK3 targeted shRNAs strongly inhibited expression of GluK2/3 in the hippocampal cultures (left). On the right hand side, a strong inhibition of GluK5 by the target shRNA but not with non-target or GluK4 targeted shRNAs is shown.

To study the role of axonal KARs in synaptogenesis, we used a recently developed microfluidic culture chamber where two neuronal populations are grown in isolation but connected by narrow (7.5 μm) tunnels allowing axon growth (Taylor and Jeon, 2010; Jokinen et al., 2013), (**Figure 1A**). Under these culture conditions, primary hippocampal neurons strongly expressed the mRNA for KAR subunits GluK2, GluK4, and GluK5, while GluK1 and GluK3 mRNAs were detected at lower levels both at DIV7 and at DIV14 (**Figure 1B**). This expression profile was asymmetrically controlled in the two-chamber microfluidic device using lentiviral vectors encoding various epitope tagged KAR subunits (**Table 2**) or shRNA against the endogenously expressed subunits GluK2 and GluK5. The ability of the shRNAs to suppress expression of the target subunits in hippocampal neurons was validated using western blot (**Figure 1C**). Axons expressing recombinant KAR subunits were identified with immunostaining against myc or flag, while the shRNA constructs contained EGFP. Co-immunostaining with antibodies against phospho-Tau(Ser396), synaptophysin and PSD-95 to visualize the axon body, pre- and post-synaptic structures, respectively, enabled analysis of the effects of various KAR subunits on synaptic development and differentiation (**Figure 1A**).

**FIGURE 2:**
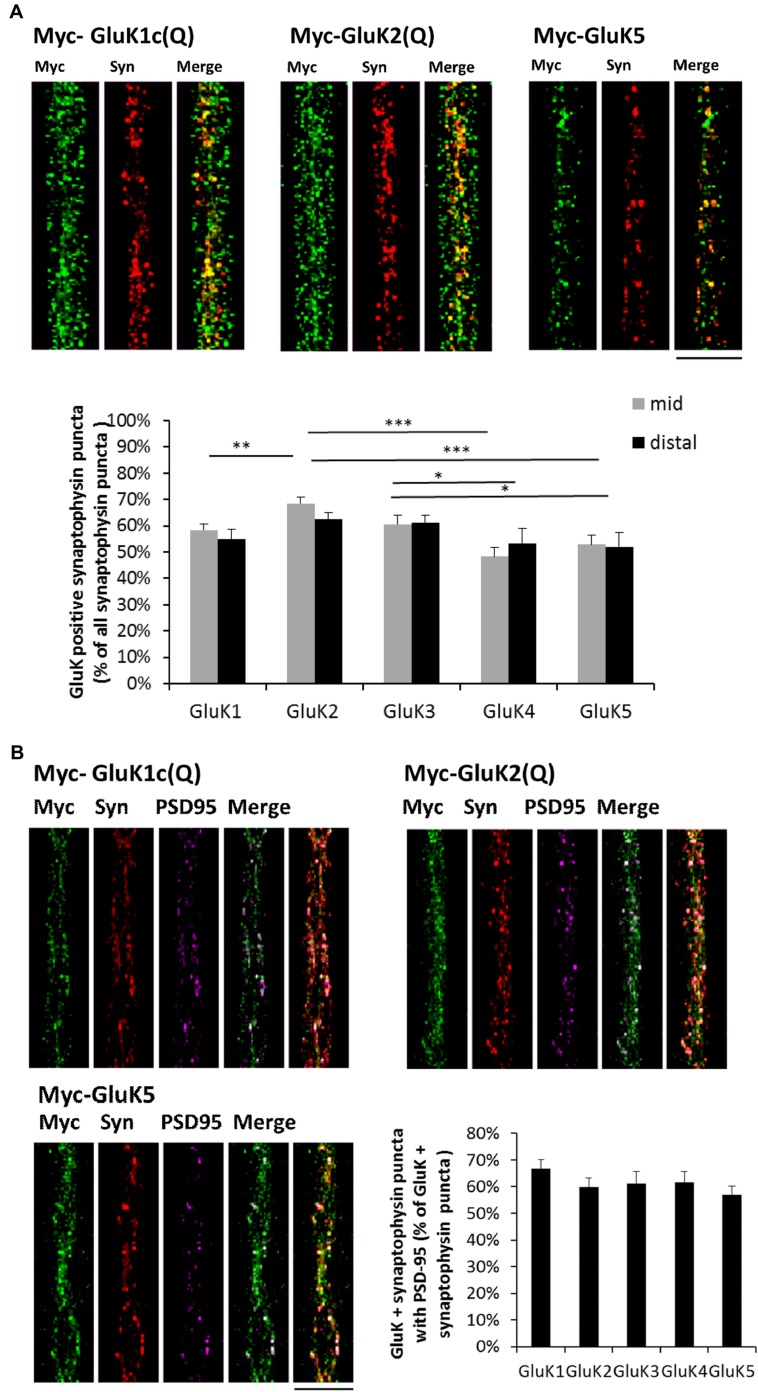
**Recombinant GluK subunits co-localize with synaptophysin independently of dendritic contact. (A)** Example images depicting synaptophysin (red) and myc-GluK (green) immunostaining in isolated axons in the mid part of the tunnels. Merged images are shown in the right panel. Pooled data on the percentage of GluK positive synaptophysin puncta/total synaptophysin puncta for myc-GluK1c(Q) (*n* = 27, 27), myc-GluK2(Q) (*n* = 24, 27), myc-GluK3(Q) (*n* = 16, 17), myc-GluK4 (*n* = 16, 18), and myc-GluK5 (*n* = 21, 14) subunits in mid an distal parts of the microfluidic tunnels. ^∗^*p* < 0.05; ^∗∗^*p* < 0.01; ^∗∗∗^*p* < 0.005. Scale bar 15 μm. **(B)** Example images illustrating synaptophysin (red), myc-GluK (green) and PSD-95 (magenta) triple staining in distal parts of the tunnels. Merged images of green + magenta and red +green + magenta are shown. Pooled data on the percentage of GluK positive synaptophysin puncta with PSD-95 contact/total GluK + synaptophysin puncta for myc-GluK1c(Q) (*n* = 36), myc-GluK2(Q) (*n* = 39), myc-GluK3(Q) (*n* = 28), myc-GluK4 (*n* = 27), and myc-GluK5 (*n* = 32). Scale bar 15 μm.

The mid part of the microfluidic tunnels lacks dendrites, but contains isolated axons that bundle together. In the distal part, the axons crossing the tunnels form synaptic contacts with the wild-type dendrites, growing <250 μm into the microgrooves from the opposing side of the culture chamber (Jokinen et al., 2013; **Figure 1A**). The recombinant KAR subunits were detected in axons and localized prominently in axonal protrusions as reported previously (Vesikansa et al., 2012). All the recombinant GluK subunits co-localized with synaptophysin (Pearson’s correlation coefficient for GluK synaptophysin co-localization: GluK1 0,60; GluK2 0,61; GluK3 0,56; GluK4 0,47; GluK5 0,63), which was detected in a subpopulation of the GluK positive axonal protrusions. On average, GluK2 and GluK3 were detected in synaptophysin positive puncta more frequently (66 ± 2 and 62 ± 2% of synaptophysin puncta, respectively) as compared to GluK1 (56 ± 2%), GluK4 (51 ± 3%) or GluK5 (53 ± 3%). Interestingly, this percentage was not different between the middle and distal parts of the tunnels for any of the subunits, suggesting that localization of recombinant GluK subunits to axonal release sites was not significantly affected by dendritic contact (**Figure 2A**). PSD-95 co-immunostaining revealed that in the distal regions of the tunnels, an average of 61 ± 2% of the GluK positive synaptophysin puncta had dendritic contact, with no significant difference between the subunits (*p* = 0.33, **Figure 2B**).

### Axonal KARs Regulate the Density of Synaptophysin Positive Vesicle Clusters

**FIGURE 3:**
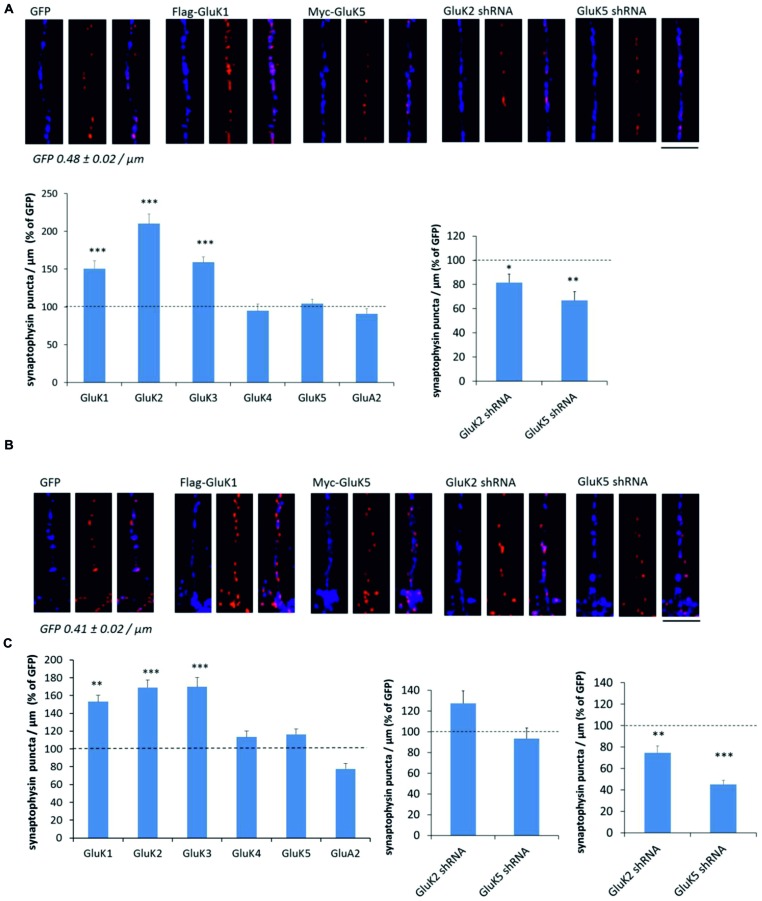
**KARs promote presynaptic differentiation by enhanced clustering of synaptic vesicles. (A)** Example images depicting synaptophysin (red) and phosphoTau (blue) immunostaining in axons expressing GFP, GluK1, and GluK5 as well as GluK2 and GluK5 shRNA in the mid part of the tunnels (top). Pooled data on the density of synaptophysin positive puncta in axons expressing various kainate receptor subunits (flag-GluK1 *n* = 123; myc-GluK2 *n* = 77; myc-GluK3 *n* = 42; myc-GluK4 *n* = 47; myc-GluK5 *n* = 100) or the AMPA subunit myc-GluA2 (*n* = 85). The effect of GluK2 shRNA (*n* = 111) and GluK5 shRNA (*n* = 94) on the density of synaptophysin positive clusters is shown on the right. The data is expressed as percentage of level of control (GFP or mock shRNA expressing) axons within the same culture batch. ^∗^*p* < 0.05; ^∗∗^*p* < 0.01; ^∗∗∗^*p* < 0.005 as compared to control. Scale bar 12 μm. **(B)** Corresponding data as in **(A)**, from the distal part of the tunnels where the infected axons make synaptic contact to wild-type dendrites. flag-GluK1 *n* = 140; myc-GluK2 *n* = 76; myc-GluK3 *n* = 48; myc-GluK4 *n* = 50; myc-GluK5 *n* = 83; myc-GluA2 *n* = 94; GluK2 shRNA *n* = 115; GluK5 shRNA *n* = 98. Scale bar 12 μm. **(C)** The effect of GluK2 shRNA (*n* = 95) and GluK5 shRNA (*n* = 102) on the density of synaptophysin positive clusters in the proximal part of the tunnels. ^∗∗^*p* < 0.01; ^∗∗∗^*p* < 0.005 as compared to control.

To study the effect of KARs on presynaptic differentiation, we used synaptophysin immunostaining to visualize synaptic vesicle clusters in the axons where KAR expression was manipulated using lentiviral vectors. In isolated axons in the middle of the tunnels, expression of GluK1, GluK2, and GluK3 produced a robust increase in the number of synaptophysin positive puncta per axon length, while the high-affinity recombinant subunits GluK4 and GluK5 and the AMPA receptor subunit GluA2 had no effect (**Figure 3A**). Consistently, silencing the expression of endogenous GluK2 significantly reduced the density of synaptophysin puncta (**Figure 3A**). Knock-down of GluK5 also caused a strong reduction in synaptophysin positive clusters as compared to controls, which is in apparent contrast with the result showing no effect of GluK5 overexpression. However, high-affinity subunits do not form functional receptors on their own but are incorporated in heteromeric complexes with GluK1–3. The endogenous expression level of endogenous GluK5 in our culture conditions was high (**Figure 1B**), possibly preventing any further effects of overexpression of this subunit.

Similar analysis was done on distal end of the tunnels, where transduced axons form synaptic contacts with the wild-type postsynaptic neurons. As in isolated axons, expression of GluK1, GluK2, and GluK3 produced a robust increase in the density of synaptophysin puncta, while GluK4, GluK5, and GluA2 had no significant effect (**Figure 3B**). However, in contrast to isolated axons, silencing the endogenous GluK2 or GluK5 had no effect on the synaptophysin staining (**Figure 3B**).

One possibility to explain this result is that endogenous KARs are not efficiently targeted to distal axons in culture. Alternatively, presynaptic regulation by KARs might be diminished upon establishment of the dendritic contact and synapse maturation. To gain insight into these possibilities, we also analyzed the effect of GluK2/5 shRNA on synaptophysin density in the proximal parts of the tunnels where axons make contact to dendrites originating from the same side of the chamber. In this part, both the GluK2 and GluK5 shRNA significantly reduced the density of synaptophysin puncta (**Figure 3C**). These data support the view that the shRNA has no effect in the distal axons, because endogenous GluK2 and GluK5 KARs are sparsely targeted there under the present culture conditions (see also Vesikansa et al., 2012). Accordingly, the density of synaptophysin puncta was significantly lower in the distal (0.41 ± 0.02/μm) as compared to proximal (0.56 ± 0.02/μm) tunnel compartments in GFP expressing axons. However, we cannot exclude the possible contribution of postsynaptic KARs, also affected by the shRNA treatment in the proximal part of the tunnel.

Together, these data suggest that axonal KAR subunits promote presynaptic differentiation leading to increase in synaptic density. This effect is seen in isolated axons, indicating that the mechanism is independent on dendritic contact.

### The Effect of KARs on Synaptophysin Puncta Depends on Channel Activity, Calcium Permeability, and Downstream Kinase Signaling

In order to understand whether the receptor activity or calcium permeability was important for the observed effects, we compared the effects of the calcium permeable (Q) and impermeable (R) editing variants of GluK1 as well as added the GluK1 selective antagonist ACET (200 nM; Dargan et al., 2009) into the culture medium of GluK1(Q) expressing neurons (DIV 3 onward).

**FIGURE 4:**
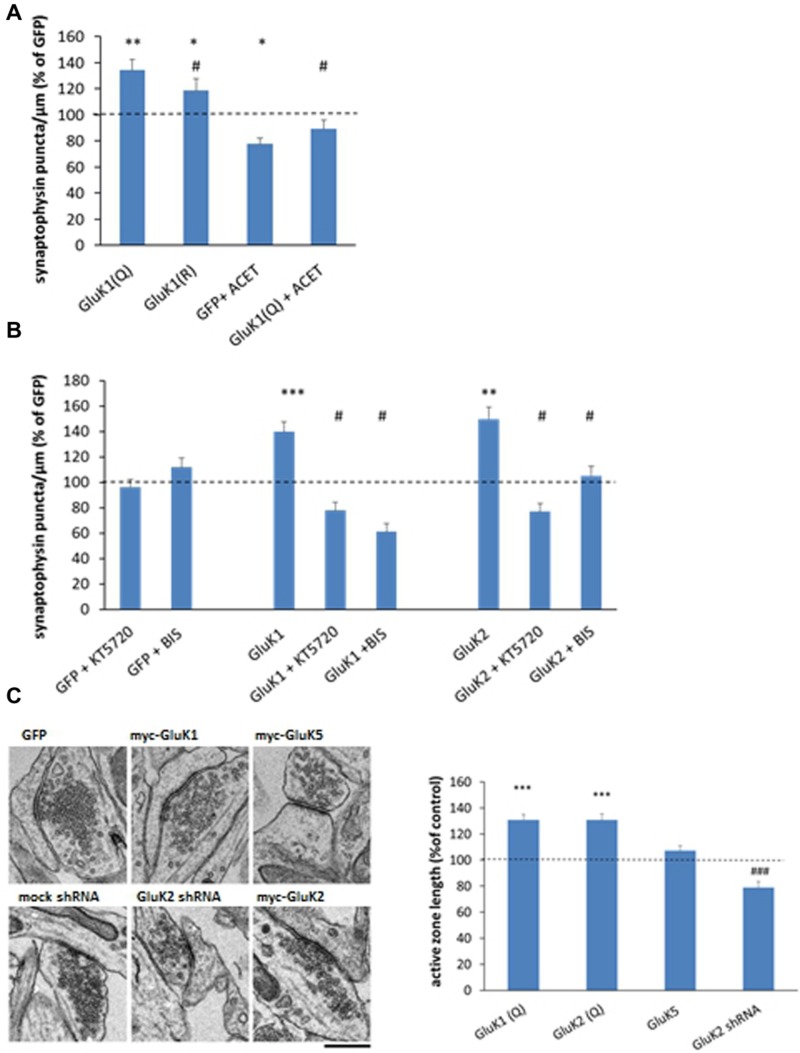
**Mechanisms underlying the effects of KARs on synaptic vesicle clustering. (A)** Averaged data comparing the calcium permeable (Q) and impermeable (R) editing variants of the GluK1 subunit as well as the effect of GluK1 selective antagonist ACET (200 nM, DIV3 onward) on synaptophysin immunostaining in isolated axons. myc-GluK1(Q) *n* = 161, myc-GluK1(R) *n* = 67; GFP + ACET *n* = 74; myc-GluK1 + ACET *n* = 82. ^∗^*p* < 0.05; ^∗∗^*p* < 0.01; ^∗∗∗^*p* < 0.005 as compared to GFP; ^#^*p* < 0.05 as compared to GluK1. **(B)** Pooled data on the synaptophysin positive puncta in the mid part of the tunnel under various experimental conditions. The data for pharmacological treatments (GFP + ACET/KT5720/BIS) represents the percentage of level of control (GFP expressing) axons within the same culture batch. The data for KAR expressing axons is normalized to level of GFP expressing sister cultures with the corresponding drug. *n* between 62 and 108 for each group. ^∗^*p* < 0.05; ^∗∗^*p* < 0.01; ^∗∗∗^*p* < 0.005 as compared to GFP; ^#^*p* < 0.05 as compared to the corresponding KAR subunit without the drug. **(C)** Electron micrographs on synaptic ultrastructure in cultured hippocampal neurons expressing GFP, GluK1, or GluK5, or after silencing endogenous GluK2 expression with shRNA. Pooled data on the average length of active zone in myc-GluK1(Q) (*n* = 168), myc-GluK2 (*n* = 263), and myc-GluK5 (*n* = 255) expressing neurons, expressed as percentage of the level at GFP infected neurons (0.40 ± 0.01 μm; *n* = 263). The data on GluK2 shRNA (*n* = 359) is normalized to the level in mock-shRNA infected neurons (0.50 ± 0.02 μm, *n* = 199). *n* refers to the number analyzed synapses. Scale bar 0.45 μm. ^∗∗∗^*p* < 0.005 as compared to GFP; ^###^*p* < 0.005 as compared to mock shRNA.

Application of ACET reduced the density of synaptophysin positive puncta in control (GFP expressing) cultures and fully blocked the effect of GluK1 overexpression (**Figure 4A**), indicating that channel function was critical for the ability of KARs to promote synaptic vesicle clustering. Furthermore, while expression of the calcium impermeable variant GluK1(R) produced a significant increase in the density of synaptophysin positive vesicle clusters as compared to GFP (*p* = 0.02), this effect was smaller as compared to the GluK1(Q) (*p* = 0.03) suggesting that calcium permeability of the channel contributes to the effects of GluK1 on presynaptic differentiation.

To gain insight on the downstream signaling mechanisms involved, the effects of GluK1, GluK2, and GluK5 on presynaptic differentiation were analyzed in the presence of KT5720 (1 μM) and bisindolylmaleimide VII acetate (BIS, 0.5 μM), inhibitors of protein kinase A (PKA) and C (PKC), respectively. Inclusion of these inhibitors in the culture medium (DIV4-) had no apparent effect on the synaptophysin puncta in GFP expressing axons, but fully prevented or reversed the effects of GluK1 and GluK2 (**Figure 4B**).

Finally, analysis of transmission electron micrographs revealed that expression of the low-affinity subunits [GluK2, GluK1] but not the high-affinity subunit GluK5, was associated with significant widening of the synaptic active zone (**Figure 4C**). Consistently, the active zone length was smaller in the cultures where endogenous GluK2 KARs was silenced, suggesting that KAR expression also affected synaptic ultrastructure.

### Facilitation and Inhibition of Presynaptic Efficacy by Low- and High-Affinity KARs

**FIGURE 5:**
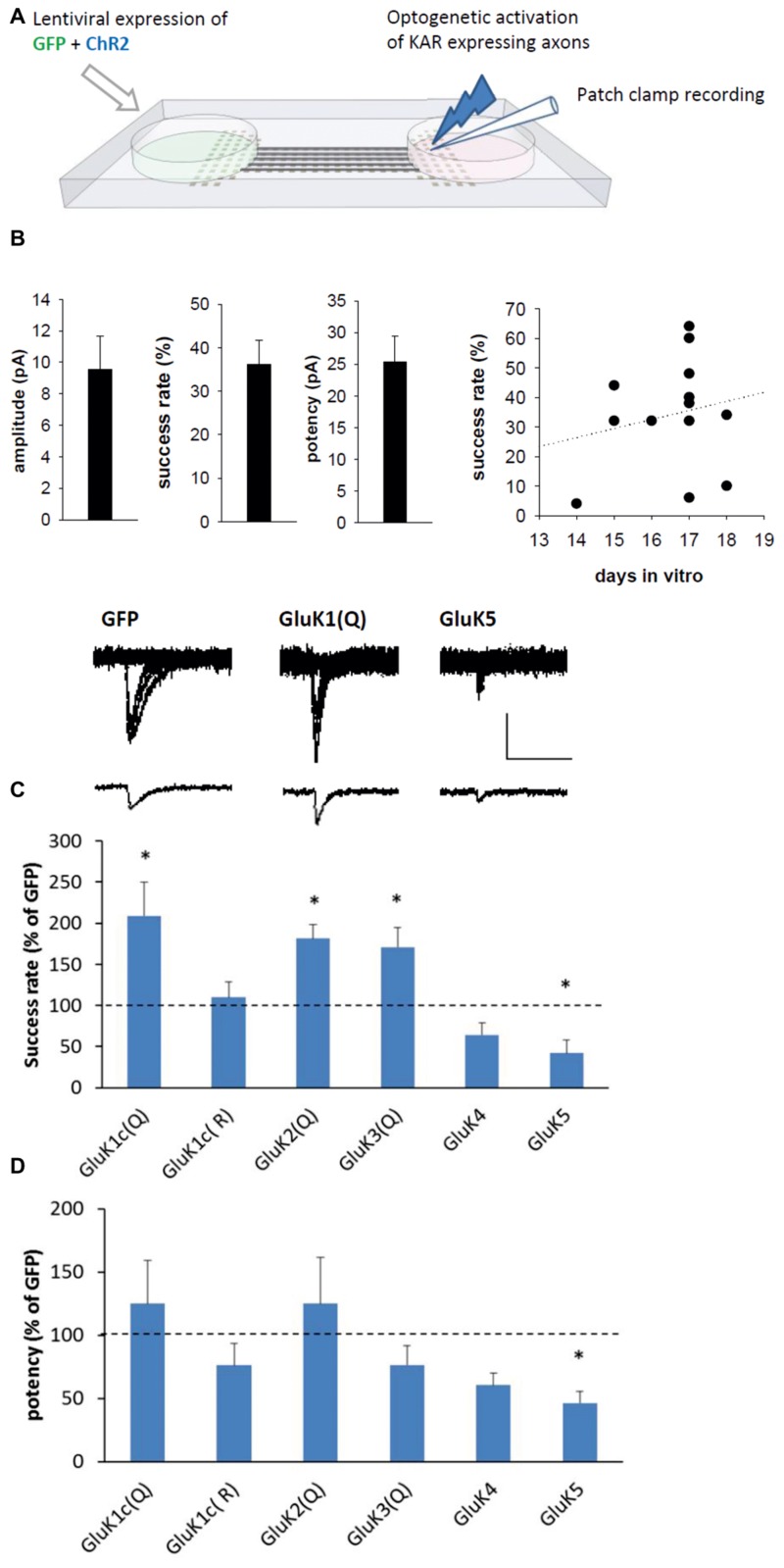
**Subtype-specific effect of KARs on presynaptic function. (A)** The experimental system to study synaptic transmission with selective presynaptic manipulation of KAR expression. Neurons on one side of the microfluidic chamber were co-transduced with various KAR subunits and channelrhodopsin (ChR2). The KAR expressing axons crossing the tunnels to the other side were selectively excited with blue light and postsynaptic responses were recorded under voltage clamp from wild-type neurons. **(B)** Pooled data on the average amplitude, success rate, and potency of recorded postsynaptic currents in the GFP expressing cultures (*n* = 13). As the success rate varied between culture batches and with the time *in vitro* (DIV; scatter plot on the right), the data on KAR expressing axons is normalized to corresponding controls. **(C)** Example traces (top) of light-evoked currents in synapses expressing GFP, GluK1(Q), and GluK5 presynaptically. The bar graph depicts pooled data on the success rate for postsynaptic currents (% of GFP) when various KAR subunits are presynaptically expressed. myc-GluK1(Q) *n* = 8, myc-GluK1(R) *n* = 7, myc-GluK2 *n* = 13, myc-GluK3 *n* = 7, myc-GluK4 *n* = 7, myc-GluK5 *n* = 6. ^∗^denotes statistical significance (*p* < 0.05) compared to GFP. Scale 20 pA, 50 ms. **(D)** Pooled data for the potency of postsynaptic currents, from the same data as in **(C)**.

The observed effects of KARs on synaptic structure would be expected to influence synaptic function. To study their effects on synaptic transmission, various KAR subunits were co-expressed with channel rhodopsin 2 [ChR2(H134R); Boyden et al., 2005] in one side of the microfluidic chamber. Co-expression of ChR2 makes the KAR expressing axons crossing the tunnels sensitive to light induced excitation, and thus allows their selective stimulation while recording postsynaptic responses from wild-type neurons (**Figure 5A**).

Light-induced postsynaptic responses were recorded from the wild-type neurons at 14–18 DIV. In the GFP/ChR2 expressing synapses, the average success rate of the responses was 34 ± 6%, with an average amplitude of 11 ± 3 pA and potency of 28 ± 5 pA (*n* = 13; **Figure 5B**). No paired-pulse facilitation of the responses were detected (ratio of second/first pulse amplitude 0.96 ± 0.24, with inter-pulse interval of 100 ms). Since the properties of transmission varied between culture batches and during development, the data on KAR expressing neurons is normalized to the level in corresponding GFP expressing cultures. Presynaptic expression of the GluK1–GluK3 subunits (Q editing variants) strongly increased the success rate of the light-evoked responses without significantly affecting the potency (**Figure 5C**), an effect consistent with an increase in the probability of glutamate release (Pr). Expression of the calcium-impermeable GluK1(R) editing variant had no effect on transmission, suggesting that the effects of GluK1–3 on Pr are due to their calcium permeability. In contrast, the success rate was significantly reduced in synapses where the high-affinity subunits GluK4 and GluK5 were presynaptically expressed (**Figure 5C**). Presynaptic expression of GluK5 but not GluK4 also significantly reduced the potency of the responses (*p* = 0.02; **Figure 5D**).

## Discussion

Kainate type of glutamate receptors have been implicated in a variety of physiological processes both in developing and adult brain (Jane et al., 2009; Contractor et al., 2011; Lerma and Marques, 2013). However, their exact roles in regulating presynaptic processes remain controversial, due to lack of specific antibodies and pharmacological tools against different types of KARs and in particular, because of indirect methodological approaches to address presynaptic function. To circumvent these problems, we have here used a microfluidic system (Jokinen et al., 2013) that allows specific manipulation of KAR expression in presynaptic neurons to study their specific roles synaptic development and function *in vitro*. Using this novel technology, we found that axonal calcium-permeable KARs promote the strength of the efferent neuronal connection, via influencing morphological differentiation and transmission efficacy.

All the recombinant KAR subunits were detected in axons and co-localized with synaptophysin independently of dendritic contact. Expression of the low-affinity (GluK1–3) but not high-affinity (GluK4,5) KAR subunits was associated with a large increase in density of synaptic vesicle clusters in isolated axons. The use of shRNA to knockdown endogenous expression of GluK2 and GluK5, the most prominent endogenously expressed subunits, confirmed a role for native GluK2 and also suggested a role for GluK5 in presynaptic differentiation. GluK4 and GluK5 do not form homomeric receptors and are functional only upon heteromerization with GluK1–3, which together with the high endogenous expression level, likely explains the lack of effect of overexpression of these subunits. As recent data indicates that GluK5 influences surface expression and synaptic targeting of GluK2 (Fernandes et al., 2009; Vesikansa et al., 2012; Fisher and Housley, 2013; Palacios-Filardo et al., 2014), it is also possible that silencing GluK5 acts indirectly by inhibiting the function of endogenous GluK2.

The mechanism of KAR dependent presynaptic differentiation was studied using recombinant GluK1. The endogenous expression of this subunit in our culture conditions was low, thus providing a good background to study the consequences of overexpression. The effects of GluK1 on presynaptic vesicle clustering were completely blocked by selective antagonist, ACET, and significantly attenuated with the calcium-impermeable editing variant GluK1(R). These data indicate that the KAR dependent presynaptic differentiation required active calcium dependent signaling, initiated by the ionotropic receptor activity and possibly boosted by calcium influx from internal sources (e.g., Lauri et al., 2003). In addition, the effect of KARs on synaptophysin puncta was fully blocked by inhibition of PKA or PKC, implicated in the G-protein coupled signaling of KARs. Thus, our data do not allow clear-cut distinction between ionotropic and metabotropic KAR activity in the regulation of presynaptic differentiation but suggest contribution of both.

What could be the possible mechanism underlying these effects? As the effects of KARs on synaptophysin puncta were dependent on receptor activation and consequently, on the presence of glutamate in the extracellular space, KAR most likely acted by stabilizing immature sites were already releasing glutamate. Such functional release sites can form in the absence of local dendritic contacts (Matteoli et al., 1992; Kraszewski et al., 1995; Krueger et al., 2003) and share the same exocytic machinery and mechanisms of endocytic recycling as mature synaptic sites (Krueger et al., 2003). Calcium permeable kainate receptors have been shown to block motility of axonal filopodia or growth cones (Tashiro et al., 2003; Ibarretxe et al., 2007; Marques et al., 2013). This motility block has been suggested to stabilize a nascent contact and to promote differentiation from filopodia to a mature synapse (Tashiro et al., 2003). Thus, stabilization of filopodia due to elevated ionotropic KAR signaling could contribute to the observed increase of synaptophysin immunopositive puncta in the axons expressing low-affinity, calcium permeable KAR subunits.

Presynaptic differentiation requires assembly of scaffolding molecules and subsequently, synaptic vesicles to the immature terminal. G-protein and PKA dependent phosphorylation of synapsin I is shown to influence distribution of synaptic vesicles in the growth cone in response to KAR activation (Gelsomino et al., 2013), providing evidence for direct KAR mediated signaling in regulation of presynaptic assembly. In addition, KARs may interact and regulate transmembrane protein complexes involved in presynaptic differentiation. In microfluidically isolated axons lacking dendritic contact, heterophilic signals from the postsynaptic neurons are absent although molecular interactions with neighboring axons are plausible. Recently GluK1 and GluK2 subunits have been shown to interact with neuropilin- and tolloid-like 1 and 2 (NETO1/2), implicated in postsynaptic scaffolding in the drosophila neuromuscular junction (Kim et al., 2012). In addition, KARs are reported to associate with cadherin catenin complexes (Coussen et al., 2002), which are capable of homophophilic interaction and have established function in synaptic differentiation (Arikkath and Reichardt, 2008).

The structural effects of KAR on connectivity were reinforced by their effects on presynaptic function. Thus, presynaptic expression of the low-affinity calcium permeable KARs [GluK1(Q), GluK2(Q), GluK3(Q)] led to a robust increase in the presynaptic efficacy, manifested as a high Pr and associated with widening of the synaptic active zone. Interestingly, presynaptic calcium permeable KAR are highly expressed at the hippocampal mossy fiber synapse (Kamiya et al., 2002; Lauri et al., 2003; Nisticò et al., 2011), an unusually large synapse with enlarged presynaptic terminal. Consistent with the present data suggesting that presynaptic calcium-permeable KARs promote clustering of synaptic vesicles, widening of the active zone and strengthening of release probability, the functional and structural maturation of the mossy fiber synapse is impaired in GluK2 deficient mice (Marchal and Mulle, 2004; Lanore et al., 2012).

In contrast, presynaptic expression of the high-affinity subunits (GluK4, GluK5) in the microfluidic culture system was associated with a low Pr. These subunits had no detectable effect on the density of synaptophysin puncta or on the active zone length, however, functional analysis indicated reduced success rate and potency of EPSCs. Such functional changes might be mediated by molecular changes in the presynaptic release machinery, which would remain undetectable with the present analysis tools. Alternatively, it is possible that the functional effects detected in GluK5 expressing neurons are due to acute or tonic KAR mediated inhibition of transmitter release. Inhibitory presynaptic KARs have been described in various areas of the brain and in particular, at immature CA3–CA1 synapses with a low Pr (e.g., Lauri et al., 2006; Vesikansa et al., 2012). An intriguing possibility is that the inclusion of the high-affinity subunits to the receptor complex will alter their signaling properties and switch their action from facilitatory to inhibitory on transmitter release.

## Conclusion

These data suggest that expression of KARs in the presynaptic neuron is a causative factor defining the presynaptic phenotype during morphological and functional development of the synaptic connectivity. Our data supports that low affinity, calcium permeable GluK’s promote formation of high-Pr synapses, while the presence of the low-affinity GluK subunits and in particular GluK5, contribute to low-Pr.

## Author Contributions

PS performed most of the experiments. PS, AV, EO, HH, and SL were involved in conception and design of work. AV and EO made the plasmid constructs. JH, SF, and VJ manufactured the microfluidic chambers. TK-L validated the shRNA constructs. AS did the RT-PCR analysis. PS and SL analyzed the data and wrote the manuscript. All authors have read and approved the final manuscript.

## Conflict of Interest Statement

The authors declare that the research was conducted in the absence of any commercial or financial relationships that could be construed as a potential conflict of interest. HH is an employee and shareholder of Herantis Pharma Plc, which is unrelated to this study.
